# Retraction: Neuronal Differentiation Dictates Estrogen-Dependent Survival and ERK1/2 Kinetic by Means of Caveolin-1

**DOI:** 10.1371/journal.pone.0224493

**Published:** 2019-10-23

**Authors:** The PLOS ONE Editors

After publication of this article [1], concerns were raised about results reported in Figures 1, 4, and 5:

- In Figure 1A, there appears to be an image splicing line between lanes 2 and 3 of the β-actin blot.
- In Figure 4C, the P-ERK1/2 and ERK1/2 panels do not include the same numbers of lanes, and similarities were noted between data shown in lanes 1 and lane 7 of the P-ERK1/2 blot. There also appear to be vertical discontinuities in Figure 4C, P-ERK1/2 panel after lanes 2 and 6.
- In the P-ERK1/2 panel of Figure 5A, similarities were noted between the data shown in lanes 1 and 2, and there appears to be a vertical discontinuity between lanes 2 and 3.
- In the ERK1/2 panel of Figure 5C, similarities were noted between the data shown in lanes 1 and 4, and there appears to be a vertical discontinuity after lane 5.
- Similarities were noted between data shown in Figure 5A, ERK1/2 panel lanes 1–5, and Figure 5C, ERK1/2 panel lanes 6–2.
- In Figure 5C, P-ERK1/2 panel, there appear to be vertical discontinuities after lanes 3, 4, and 5, and the background appears notably different in lanes 5 and 6 compared to lanes 1–3.

For Figure 1, the authors noted that the A1 and mesPC data were obtained using different gels/blots, and that the corresponding experimental and β-actin data were obtained from the same blot in each case.

The authors provided raw image data to support the Figure 4 results. These data clarified that lanes were rearranged in preparing the ERK1/2 panel in Figure 4A and the P-ERK1/2 panels in Figure 4A and 4C, and that lane 7 in each of these panels should have been labelled as exposed to both estradiol (E_2_) and ICI 182–780. For the ERK1/2 panel of Figure 4C, the E_2_ + ICI 182–780 data are in the third lane, an additional timepoint (20 min) was included in error as the fourth lane, and lanes 5–8 are the data corresponding to the lane 3–6 labels. The raw data indicated that the wrong control data (control without ethanol) were included in lane 1 of the P-ERK1/2 panels of Figure 4A and 4C. The authors commented that similarities between lanes 1 and 7 of the P-ERK1/2 panel in Figure 4C may reflect similarities in expression levels in the two samples, or could be due to an error in the figure preparation. Per our editorial assessment the pixel data in these lanes are more similar than would be expected for different data, and the E_2_ + ICI 182–780 data on the raw blot image did not appear to match the data in the published figure. Hence, concerns about the lane 7 P-ERK1/2 data in Figure 4C remain unresolved.

For Figure 5, the authors provided quantification data for the panels in question and claimed that the data raised as similar were not the same, although per our editorial assessment the pixel data in the affected lanes are more similar than would be expected for different data. The original blot images are unavailable and so we were unable to resolve the concerns.

The underlying data were not provided with this article, although the Data Availability statement reads, “All relevant data are within the paper and its Supporting Information files.” The authors commented that the underlying data are not available for Figures 1, 2A, 5, S1, S2, S3, and S6. For Figure 3, quantitative data are available but the underlying blot images are not. Underlying data supporting other results reported in the article are available from the authors, as are FACS data to support the “data not shown” statement in the Results section.

In light of the unresolved concerns about Figures 4 and 5 as well as the unavailability of some data needed to support this article’s results, the *PLOS ONE* Editors retract this article.

It was noted after publication that the Academic Editor who handled this article’s peer review was affiliated with the same institution as one of the authors. We regret that this was not identified and addressed prior to the article’s publication.

All authors disagreed with the retraction.
